# Core versus cuttings samples for geochemical and petrophysical analysis of unconventional reservoir rocks

**DOI:** 10.1038/s41598-020-64936-y

**Published:** 2020-05-13

**Authors:** Hamed Sanei, Omid H. Ardakani, Takashi Akai, Kunio Akihisa, Chunqing Jiang, James M. Wood

**Affiliations:** 1 Lithospheric Organic Carbon Group (LOC), Department of Geoscience, Aarhus University, Aarhus, Denmark; 2 Natural Resources Canada, Geological Survey of Canada, Calgary, Alberta Canada; 3 Japan Oil, Gas and Metals National Corporation (JOGMEC), Tokyo, Japan; 4 Oil Research Limited, Tokyo, Japan; 5 Calaber1 Resources, Calgary, Alberta Canada

**Keywords:** Geochemistry, Geology, Petrology, Sedimentology

## Abstract

Core samples from petroleum wells are costly to obtain, hence drill cuttings are commonly used as an alternative source of rock measurements for reservoir, basin modelling, and sedimentology studies. However, serious issues such as contamination from drilling mud, geological representativeness, and physical alteration can cast uncertainty on the results of studies based on cuttings samples. This paper provides a unique comparative study of core and cuttings samples obtained from both vertical and horizontal sections of a petroleum well drilled in the Canadian Montney tight gas siltstone reservoir to investigate the suitability of cuttings for a wide range of geochemical and petrophysical analyses. The results show that, on average, the bulk quantity of kerogen or solid bitumen measured in cuttings is comparable to that of the core samples. However, total organic carbon (TOC) measurements are influenced by oil-based drilling mud (OBM) contamination. Solvent-cleaning of cuttings has been shown to effectively remove OBM contamination in light, medium, and heavy range hydrocarbons and to produce similar kerogen/solid bitumen measurements to that of core samples. Similarly, pyrolysis methods provide an alternative to the solvent-cleaning procedure for analysis of kerogen/solid bitumen in as-received cuttings. Microscopic study substantiates the presence of significant contamination by OBM and caved organic and inorganic matter in the cuttings, which potentially influence the bulk geochemistry of the samples. Furthermore, minerals in the cuttings display induced micro-fractures due to physical impacts of the drilling process. These drilling-induced micro-fractures affect petrophysical properties by artificially enhancing the measured porosity and permeability.

## Introduction

Recent advances in horizontal drilling and hydraulic fracturing provide the opportunity for economic hydrocarbon extraction from the tight rocks such as organic-rich mudrock and fine-grained siltstone. The reservoir quality of unconventional tight mudrocks is highly influenced by the deposited minerals and organic matter (OM) macerals present as well as their alteration by diagenetic and thermal maturity processes^1–4^. Reservoir and source quality characterization of unconventional tight mudrocks is often conducted on readily available drill cuttings samples using various organic geochemical, petrographic, and petrophysical techniques. However, analytical results of cuttings are often compromised by the use of oil-based drilling mud (OBM), caved fragments from shallower strata, and physical impacts of the drilling process. While drill-core samples are preferred for rock analysis, obtaining such samples from the horizontal section of wells is difficult, and routinely collected cuttings samples^5^ are alternatively used for conducting geochemical and petrophysical analyses.

The objective of this study is to compare the organic geochemistry, petrology, and petrophysics results of core samples versus drill cuttings obtained from closely correlated depth intervals in a unique, long exploration well containing both vertical and horizontal sections drilled into the unconventional Montney siltstone reservoir (British Columbia, Canada). The results enable a close comparison between the core and cuttings samples for their overall geological representativeness as well as the development and assessment of revised analytical methodologies for drill cuttings samples.

## Results and Discussion

Three different types of samples were collected from well 03-21-080-17W6 in the British Columbia portion of the wet-gas (condensate) window of the unconventional Montney tight gas siltstone reservoir. The four sample types are (i) as-received core (ARcore) samples; (ii) solvent-cleaned core (SCcore) (ii) as-received drill cuttings (ARcutt) sieved to 1–2 mm size; and (iii) hand-sorted/picked, solvent-cleaned drill cuttings (SCcutt). The solvent-cleaning was conducted using a mixture of toluene and methanol (for 24 hours) to remove OBM contamination. The ARcore samples were obtained from the closest possible depths to those of the collected drill cuttings.

### Pyrolysis geochemistry

**Figure 1:**
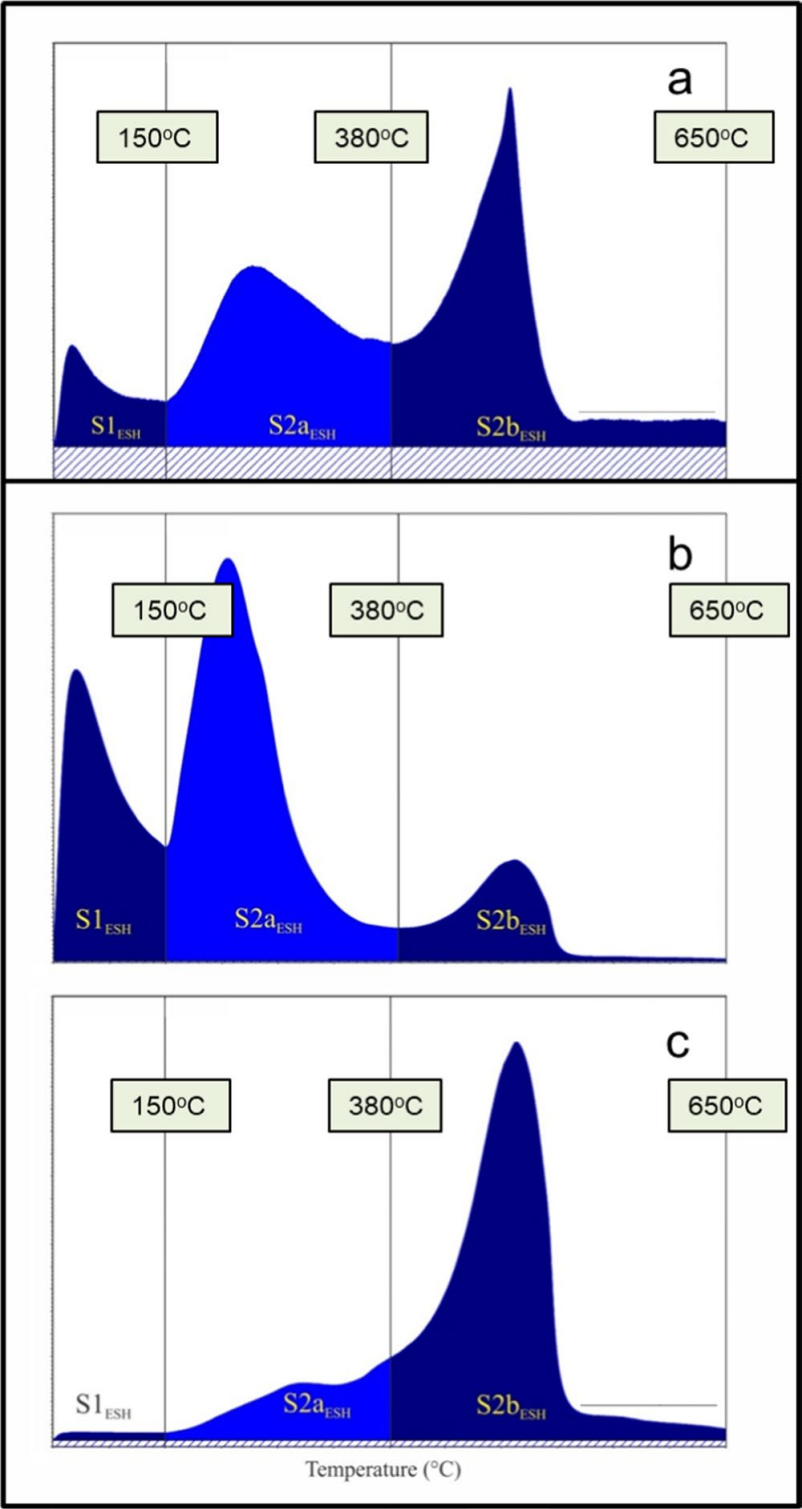
Flame Ionization Detector (FID) pyrograms showing the evolution of hydrocarbon peaks versus temperature at the initial iso-temperature of 150 °C (S1_ESH_) followed by a ramp heating of 10 °C per minute up to 650 °C. The continuous ramping heat in the extended slow heating (ESH) pyrolysis method^6,7^ evolves two distinct peaks (S2a_ESH_ and S2b_ESH_) separated at approximately 380 °C. This dividing temperature between S2a and S2b may vary depending upon the geochemical composition of the hydrocarbons in the rock. (**a**) as-received core (ARcore) from the measured depth of 2165.5 m, (**b**) as-received cuttings (ARcutt) from the measured depth of 2155 m, and (**c**) solvent-cleaned cuttings (SCcutt) from the same depth of 2155 m.

In order to better differentiate OM fractions in the samples, a manually devised extended slow heating (ESH) pyrolysis was utilized^6^. ESH pyrolysis is a modified Rock-Eval analysis with lower iso-temperature of 150 °C (10 minutes) and reduced continuous ramping temperature of 10 °C per minute (up to 650 °C) to allow for better release of volatile hydrocarbons (VHC; mg HC/g Rock) and resolution of hydrocarbon peaks from kerogen. The continuous ramping temperature allows separation between free hydrocarbons and kerogen to occur naturally. This is a distinct advantage over the standard Rock-Eval method, which forces a division between S1 and S2 peaks by holding at an iso-temperature of 300 °C. The resulting peaks in the ESH method are (i) volatile hydrocarbon (VHC) desorbed at 150 °C (S1_ESH_), (ii) fluid hydrocarbon residue (FHR), which desorbs medium to heavy free hydrocarbons released between 150 °C to approximately 380 °C (S2a_ESH_) in the continuous heating ramp of 10 °C/min, (iii) thermal cracking of hydrocarbons in kerogen/solid bitumen (S2b_ESH_) in the temperature range of ~380 to 650 °C (Fig. 1). All peaks are measured in mg HC/g rock. The quantity of S2b (mgHC/g) in the ESH method was converted to the weight percent (wt. %) and added to the residual carbon (wt.%) obtained from oxidation heating of the sample during the ESH process to calculate the quantity of the non-extractable (kerogen/bitumen) fraction of OM in the rock. Sanei *et al*.^6^ and Wood *et al*.^7^ have shown that in the British Columbia portion of the unconventional tight reservoir virtually all the kerogen in the Montney Formation consists of migrated solid bitumen. Thus, the ESH method can measure the quantity of solid bitumen in the bulk samples.

**Figure 2:**
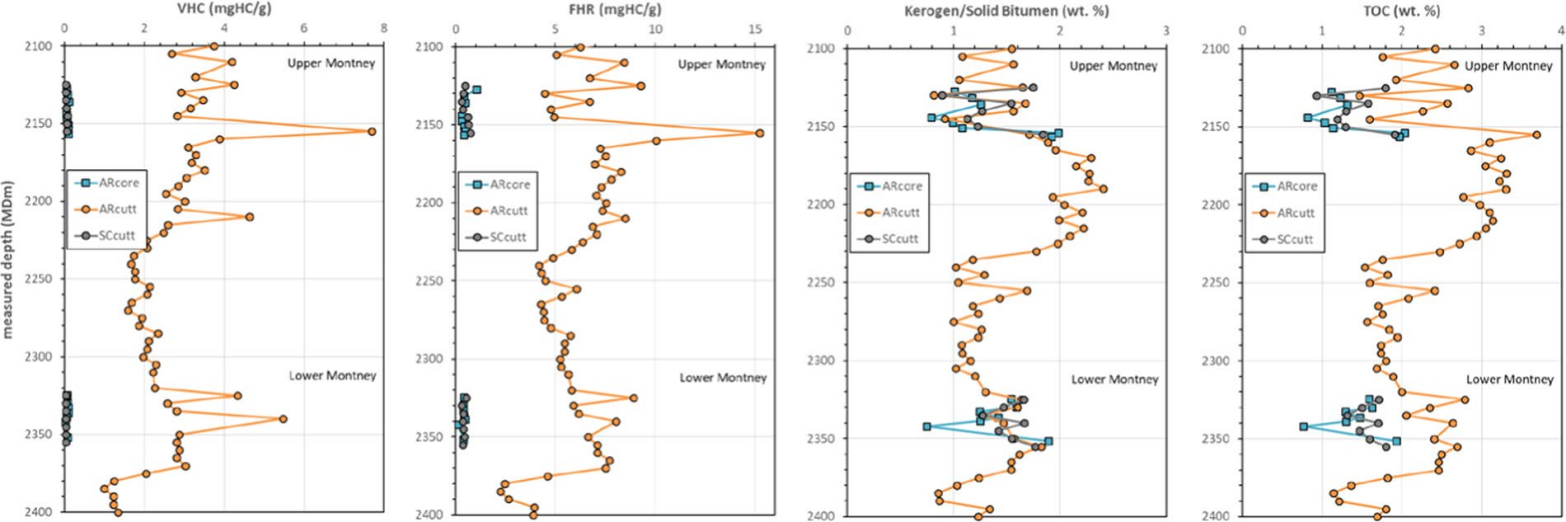
Depth profile of the organic matter (OM) fractions in the as-received core (ARcore), as-received cuttings (ARcutt), and cleaned cuttings (SCcutt) obtained from similar depths (measured depth, m) in an Upper and Lower Montney cored well, using the Extended Slow Heating (ESH) pyrolysis technique. The OM fractions include: volatile hydrocarbon (VHC; mg HC/g), fluid hydrocarbon residue (FHR; mg HC/g), kerogen/solid bitumen (wt.%), and total organic carbon (TOC, wt.%).

Results of ESH pyrolysis for the three sets of ARcore, ARcutt, and SCcutt samples obtained from the closely correlated depth intervals are presented in Table 1. The results show that the VHC fraction as represented by S1_ESH_ is significantly higher in the ARcutt (mean 3.76 mgHC/g) compared to ARcore (mean 0.07 mgHC/g) and SCcutt samples (mean 0.04 mgHC/g) (Table 1; Figs. 1, 2). This indicates that the VHC fraction in the ARcutt samples is highly enriched by OBM contamination when compared to their ARcore counterparts. This is in agreement with other studies, which reported that the VHC fraction is significantly influenced by OBM contamination in Montney siltstones^6,8^. The VHC fraction present in the ARcutt samples appears to be effectively removed by the cleaning procedure for the SCcutt samples (Fig. 1).

**Table 1:** Organic matter (OM) fractions in as-received core (ARcore), as-received cuttings (ARcutt), and solvent-cleaned cuttings (SCcutt) obtained from similar depths (measured depth, m) in an Upper and Lower Montney cored well, using the Extended Slow Heating (ESH) pyrolysis technique.

	Sample	Depth	VHC	FHR	Kerogen/ Solid Bitumen	TOC
	#	m	mg HC/g	mg HC/g	wt.%	wt.%
***As-received core (ARcore)***	CO-48	2127.45	0.05	1.04	1.01	1.11
	CO-49	2131.5	0.08	0.42	1.17	1.22
	CO-50	2135.92	0.11	0.47	1.26	1.31
	CO-51	2144.14	0.05	0.29	0.79	0.82
	CO-52	2147.28	0.06	0.31	1.00	1.03
	CO-53	2150.88	0.10	0.42	1.08	1.13
	CO-54	2153.97	0.09	0.45	1.98	2.03
	CO-55	2156.5	0.10	0.42	1.92	1.97
	CO-56	2324.83	0.07	0.43	1.54	1.59
	CO-57	2330	0.07	0.38	1.59	1.63
	CO-58	2332.72	0.10	0.38	1.25	1.29
	CO-59	2336.41	0.10	0.41	1.42	1.47
	CO-60	2338.8	0.05	0.46	1.25	1.30
	CO-61	2342.22	0.02	0.12	0.75	0.76
	CO-62	2351.69	0.07	0.38	1.89	1.93
		**mean**	**0.07**	**0.43**	**1.33**	**1.37**
***As-received cuttings (ARcutt)***	16	2125	4.25	9.28	1.65	2.83
	17	2130	2.91	4.46	0.82	1.46
	18	2135	3.46	6.72	1.68	2.57
	19	2140	3.15	4.77	1.56	2.26
	20	2145	2.82	4.95	0.92	1.59
	22	2155	7.69	15.24	1.71	3.69
	56	2325	4.33	8.93	1.63	2.79
	57	2330	2.57	5.91	1.60	2.35
	58	2335	2.80	6.17	1.27	2.05
	59	2340	5.47	8.03	1.47	2.64
	61	2350	2.88	6.64	1.57	2.40
	62	2355	2.80	7.10	1.82	2.69
		**mean**	**3.76**	**7.35**	**1.47**	**2.44**
***Solvent-Cleaned cuttings (SCcutt)***	16	2125	0.03	0.46	1.75	1.79
	17	2130	0.03	0.38	0.89	0.93
	18	2135	0.03	0.30	1.54	1.57
	19	2140	0.04	0.34	1.27	1.30
	20	2145	0.08	0.60	1.13	1.19
	21	2150	0.06	0.62	1.23	1.29
	22	2155	0.06	0.75	1.84	1.91
	56	2325	0.04	0.52	1.66	1.71
	57	2330	0.03	0.31	1.47	1.50
	58	2335	0.03	0.39	1.27	1.31
	59	2340	0.03	0.36	1.66	1.70
	60	2345	0.03	0.39	1.42	1.46
	61	2350	0.03	0.44	1.55	1.59
	62	2355	0.03	0.35	1.77	1.80
		**mean**	**0.04**	**0.44**	**1.46**	**1.50**

The OM fractions include: (i) volatile hydrocarbon (VHC) in mg HC/g, (ii) fluid hydrocarbon residue (FHR) in mg HC/g, and (iii) kerogen/solid bitumen in wt.%. Total organic carbon (TOC) content is in wt.%.

Similarly, the FHR fraction (S2a_ESH_) is highly enriched in the ARcutt samples (7.35 mgHC/g) compared to the ARcore (0.43 mgHC/g) and SCcutt samples (0.44 mgHC/g) (Table 1; Figs. 1, 2). The results show the cleaning process effectively removed almost the entire FHR fraction. The effect of OBM removal by the solvent cleaning treatment is also evident in FID pyrograms, which show removal of the S1_ESH_ in the SCcutt sample as compared to the original ARcutt sample from the same depth interval (Fig. 1). This result shows that the process of OBM removal effectively eliminated the volatile hydrocarbons (S1_ESH_) as well as most of the FHR attributed to medium to heavy range hydrocarbons (S2a_ESH_).

As demonstrated by Sanei *et al*.^6^, ESH pyrolysis is superior to standard Rock-Eval pyrolysis as it allows sufficient time for volatile free hydrocarbons to evolve. Evolution of the S1_ESH_ hydrocarbon peak at the lower iso-temperature of 150 °C over a longer initial time of 10 minutes represents the contaminants from OBM as well as a minor amount of any remaining volatile free hydrocarbon (light oil and gas condensates) trapped within the rock. The result of this study shows that the VHC in ARcore samples is negligible (0.07 mgHC/g) compared to ARcutt samples from similar depths (3.76 mgHC/g) (Table 1; Fig. 2). This indicates that most of the measured VHC in the ARcutt samples is due to OBM contamination. The VHC fraction is shown to be efficiently removed from the SCcutt samples by the cleaning procedure. At higher thermal maturity, where no indigenous volatile hydrocarbons are retained in the samples, the S1_ESH_ peak can be attributed mostly to OBM contamination.

The S2b_ESH_ fraction is attributed to the remaining hydrocarbons of the non-extractable OM, which is kerogen. In the case of Montney siltstones, virtually all the kerogen fraction consists of solid bitumen^6,9,10^. Hence, the sum of S2b_ESH_ and residual carbon (RC wt.%) gives the bulk concentration (wt.%) of the kerogen/solid bitumen fraction (wt.%). The mean kerogen/solid bitumen fraction is 1.33 wt.% in the ARcore samples and 1.47 wt.% in the ARcutt samples. The relative difference between the mean solid bitumen fraction of the ARcore and ARcutt samples is 11%, which is within the natural variability of the rocks in the studied intervals. This indicates that the solid bitumen fraction measured by the ESH pyrolysis is reliable and not significantly influenced by OBM contamination. The mean kerogen/solid bitumen fraction in the SCcutt samples (1.46 wt.%) shows a slight relative difference of ~1% from that of the ARcutt samples before the cleaning procedure (1.47 wt.%; Table 1, Fig. 2). This indicates that the cleaning procedure did not make a significant difference to the kerogen/solid bitumen fractions and further confirms that the OBM contamination did not significantly affect the kerogen/solid bitumen fractions. This also reiterates the assessment that the kerogen/solid bitumen fraction measured in the ESH pyrolysis is a robust kerogen geochemical parameter for the as-received cuttings samples because it does not appear to be influenced by the OBM contamination and hence does not require the costly solvent-cleaning procedure.

The TOC content of the ARcore samples, as measured by the standard Rock-Eval analysis, has a mean value of 1.37 wt.% (Table 1, Fig. 2). This is significantly lower (by 78% in a relative sense) than the value for SCcutt samples (2.44 wt.%, Table 1, Fig. 2). The significant enrichment of organic content in the cuttings is due to OBM contamination, previously seen by the enrichment of VHC and FHR in the ARcutt samples relative to the ARcore counterparts. The cleaning procedure is shown to effectively remove the OBM contamination from the ARCcutt samples and to produce a mean TOC content (1.5 wt.%), which is relatively close to that of ARcore (~10% relative difference) (Table 1, Fig. 2). This further shows that TOC from the standard Rock-Eval can be significantly influenced by OBM contamination in cuttings and hence it is preferable to use the kerogen/solid bitumen fraction from the ESH method instead of cleaning cuttings with solvents, which is time consuming and costly, and also has safety and environmental considerations.

In order to characterize the molecular compositions of the hydrocarbons identified in the ESH pyrolysis method, selected ARcutt and ARcore samples were subjected to online thermal desorption/pyrolysis-gas chromatography-mass spectrometry/flame ionization detection (TD/Py-GC-MS/FID) analysis. This system allows the volatile and semi-volatile hydrocarbon components to be released from any stage (i.e., temperature range) of thermal desorption and pyrolysis to be selectively analyzed online via a coupled GC-MSD/FID for their molecular composition. Two stages of sequential TD-GC-MS/FID analysis were used to fingerprint the free hydrocarbons. Subsequently, Py-GC-MS/FID analysis was used to characterize the solid organic matter in the samples. To record the light and medium to heavy hydrocarbons, a sequential thermal desorption analysis was run at 150 °C for 3 minutes followed by 380 °C for 3 minutes respectively. Lastly, pyrolysis was conducted at 650 °C for 0.4 minutes to remove the volatile components.

**Figure 3:**
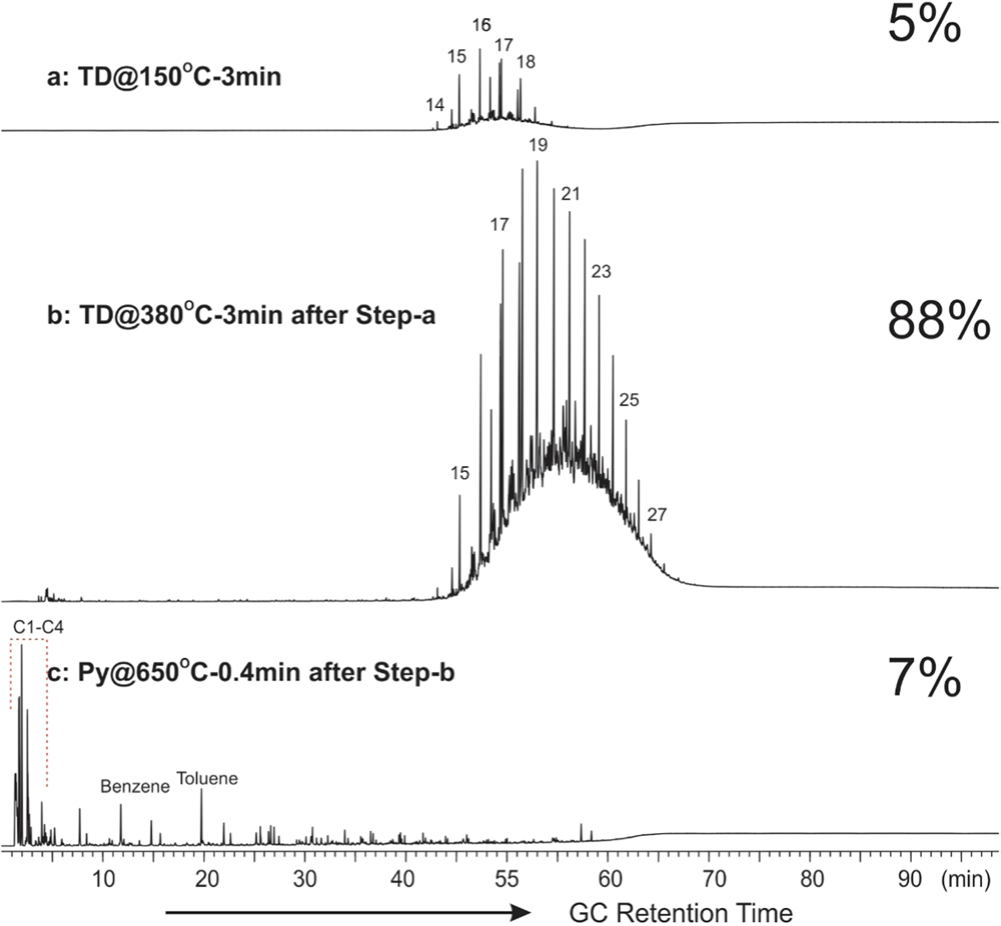
GC traces from TD/Py-GC-MS/FID analysis of a Montney cuttings sample (ARcutt) showing the composition of (**a**) free hydrocarbons thermally desorbed at 150 °C for 3 minutes; (**b**) at 380 °C for 3 minutes after thermal desorption at 150 °C; and (**c**) flash-pyrolysis at 650 °C after previous sequential thermal desorption. Numbers denote the carbon numbers of the corresponding n-alkane peaks. Percentages are the contribution of each thermal fraction to the total hydrocarbon potential of the sample.

**Figure 4:**
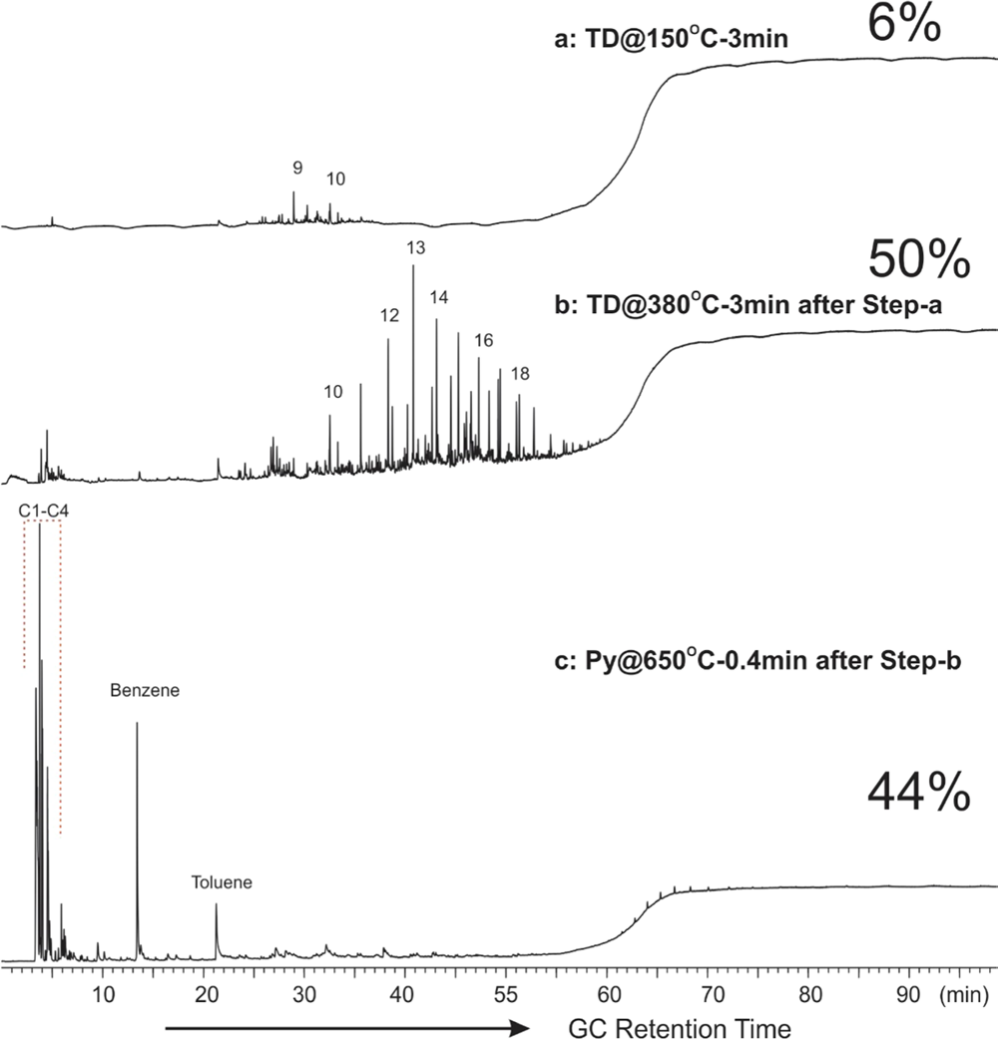
GC traces from TD/Py-GC-MS/FID analysis of a Montney core sample (ARcore) showing the composition of (**a**) free hydrocarbons thermally desorbed at 150 °C for 3 minutes; (**b**) at 380 °C for 3 minutes after thermal desorption at 150 °C; and (**c**) flash-pyrolysis at 650 °C after previous sequential thermal desorption. Numbers denote the carbon numbers of the corresponding n-alkane peaks. Percentages are the contribution of each thermal fraction to the total hydrocarbon potential of the sample.

Presented in Fig. 3 are the GC traces showing the molecular composition of free hydrocarbons desorbed at (a) 150 °C, (b) 150–380 °C, and (c) the pyrolysate from flash-pyrolysis at 650 °C for an ARcutt sample. Figure 4 presents similar GC traces for an ARcore sample collected 1.5 m below the cuttings sample. Free hydrocarbons liberated at 150 °C from the cuttings are mainly composed of C_14_ to C_19_ alkanes seated atop a minor Unresolved Complex Mixture (UCM) hump.

In comparison, hydrocarbons released between 150 and 380 °C are heavier, in the range of C_15_ to C_27_, with a maximum at C_19_ and seated atop a large UCM hump. Free hydrocarbons thermally desorbed from the ARcutt sample as well as from the ARcore sample (Figs. 3, 4) display a different fingerprint than that of the natural free hydrocarbons. The free hydrocarbons represented by the VHC fraction (S1_ESH_) and the FHR fraction (S2a_ESH_) in the ARcutt samples (Fig. 2) are likely OBM contaminants and other drilling additives such as diesel. VHC in the ARcutt sample account for over 90% of the total free hydrocarbon, while contributing to just over 50% in the ARcore sample from the same interval (Figs. 2, 3).

When flash-pyrolyzed at 650 °C, the pyrolysate from the ARcore sample is dominated by C_1_–C_4_ gaseous hydrocarbon peaks, accounting for more than 40% of the total amount of GC-amenable hydrocarbons (Fig. 4c). Benzene and toluene are the next most prominent peaks, contributing approximately 6% and 5%, respectively, to the total pyrolysate hydrocarbons. With the exception of C_5_–C_6_ alkanes and alkenes, other normal alkanes and alkenes are relatively minor components of the ARcore sample (Fig. 4c). This indicates that the solid organic matter in the ARcore sample has experienced very high thermal maturity, is comprised mainly of aromatic structures with some short aliphatic chains, and has little potential for liquid hydrocarbon generation.

By comparison, the flash-pyrolysis product from the cuttings sample contains approximately 40% gaseous C_1_–C_4_ hydrocarbons (Fig. 4c). Benzene and toluene are also among the major liquid hydrocarbons, however, light to medium hydrocarbons are more abundant in the ARcutt sample than in the ARcore sample (Figs. 3, 4c). The greater presence of light to medium hydrocarbons in the ARcutt sample could be partly due to caved solid organic matter from shallower depths.

### Organic petrology

**Figure 5:**
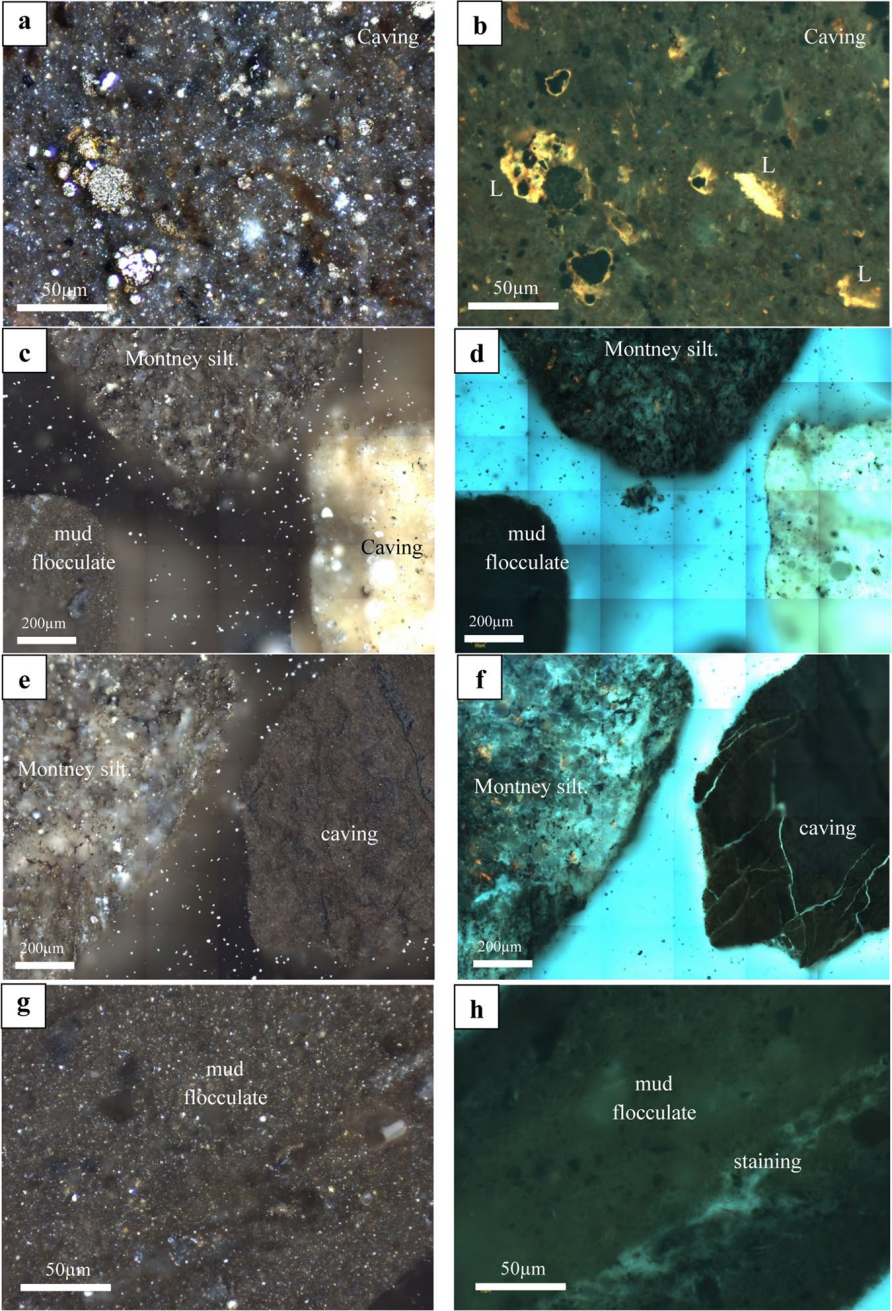
Photomicrographs showing the results of reflected light organic petrography of an as-received drill cuttings (ARcutt) sample from 2155 m depth in white light (**a,c,e,g**; images on the left column) and same view in fluorescence light (**b,d,f,h;** images on the right column). Three major sources of contamination are identified in the cuttings sample: cavings from overlying formations, clay-like matter from drilling mud, and possibly volatile, oil-based invert emulsion drilling fluids. (**a,b**) Caved fragment of organic-rich shale from overlying source rock displaying low maturity, as evidenced by the presence of brightly fluorescing liptinitic (L) particles (**b**). (**c,d**) Flocculation of drilling mud in the form of clay-like aggregates. (**e,f**) Fine-grained, shaley, organic-rich fragment, possibly caved from overlying source rocks (right side of image) shown next to the original Montney fragment (left side of image). (**g,h**) A microscopic view of Montney siltstone showing possible staining from diesel-based invert emulsion drilling fluids, apparent under fluorescence light (right image).

Organic petrology was conducted on six ARcutt samples. Results show that the cuttings have been subjected to contamination and physical deformation, which could potentially hinder the usefulness of cuttings for further reservoir characterization studies such as porosity and permeability measurements. The extreme case can be seen in the photomicrographs of a cuttings sample from 2155 m depth (Fig. 5). Three major contamination types can be observed in this sample:

(i) The occurrence of fine-grained, organic-rich, shale fragments caved from overlying source rocks, often displaying low maturity suggested by brightly fluorescing liptinitic particles (L), incompatible with the regional thermal maturity of the wet-gas window (Fig. 5a,b).
(ii) Flocculation of drilling mud in the form of clay-like aggregates (Fig. 5c,f).
(iii) Staining from diesel-based invert emulsion drilling fluids apparent under fluorescence light (Fig. 5g,h).

**Figure 6:**
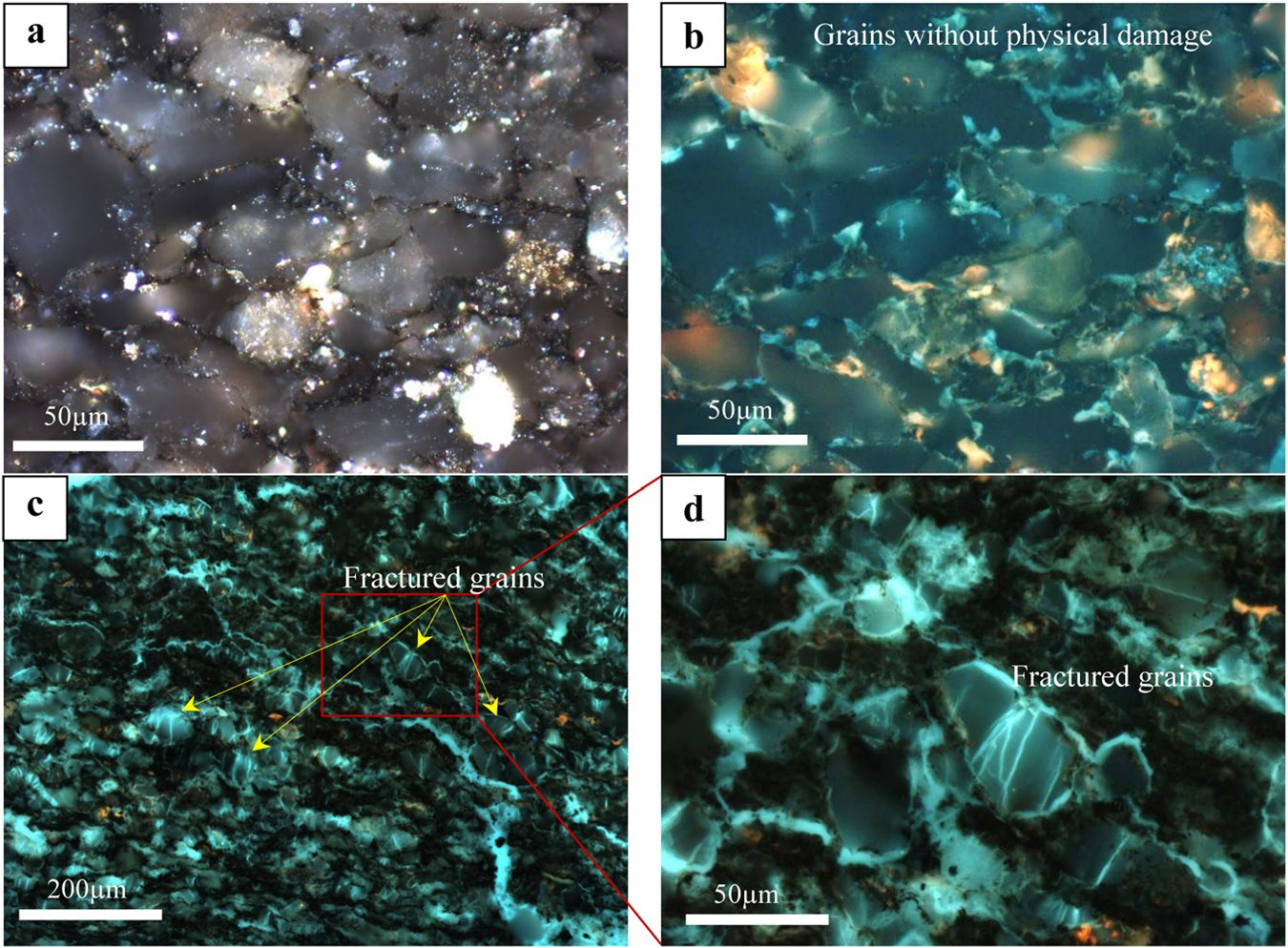
Photomicrographs showing: (**a,b**) the physical properties of the grains in the As-received core sample (ARcore) from 2155 m depth in both white **(a**) and fluorescence (**b**) reflected-light microscopy. (**c,d**) The corresponding drill cuttings (ARcutt) sample shows widespread physical deformation of the grains in the form of micro-fractures, more prevalent in the larger silt and sand grains possibly due to the stress exerted from the drilling process. These micro-fractures are absent in the corresponding ARcore samples from (**a,b**).

The petrographic results also indicate major deformation in the physical properties of the mineral grains of the ARcutt samples (Fig. 6c,d) compared to the ARcore samples (Fig. 6a,b). Photomicrographs show the occurrence of widespread micro-fractures in the silt grains of the cuttings samples. Micro-fractures are absent in the ARcore samples from similar intervals (Fig. 6a,b). These micro-fractures were likely induced by stresses exerted from the drilling process. The drilling-induced micro-fractures play a significant role in the petrophysical properties of the ARcutt samples as will be discussed next.

### Pore size

**Figure 7:**
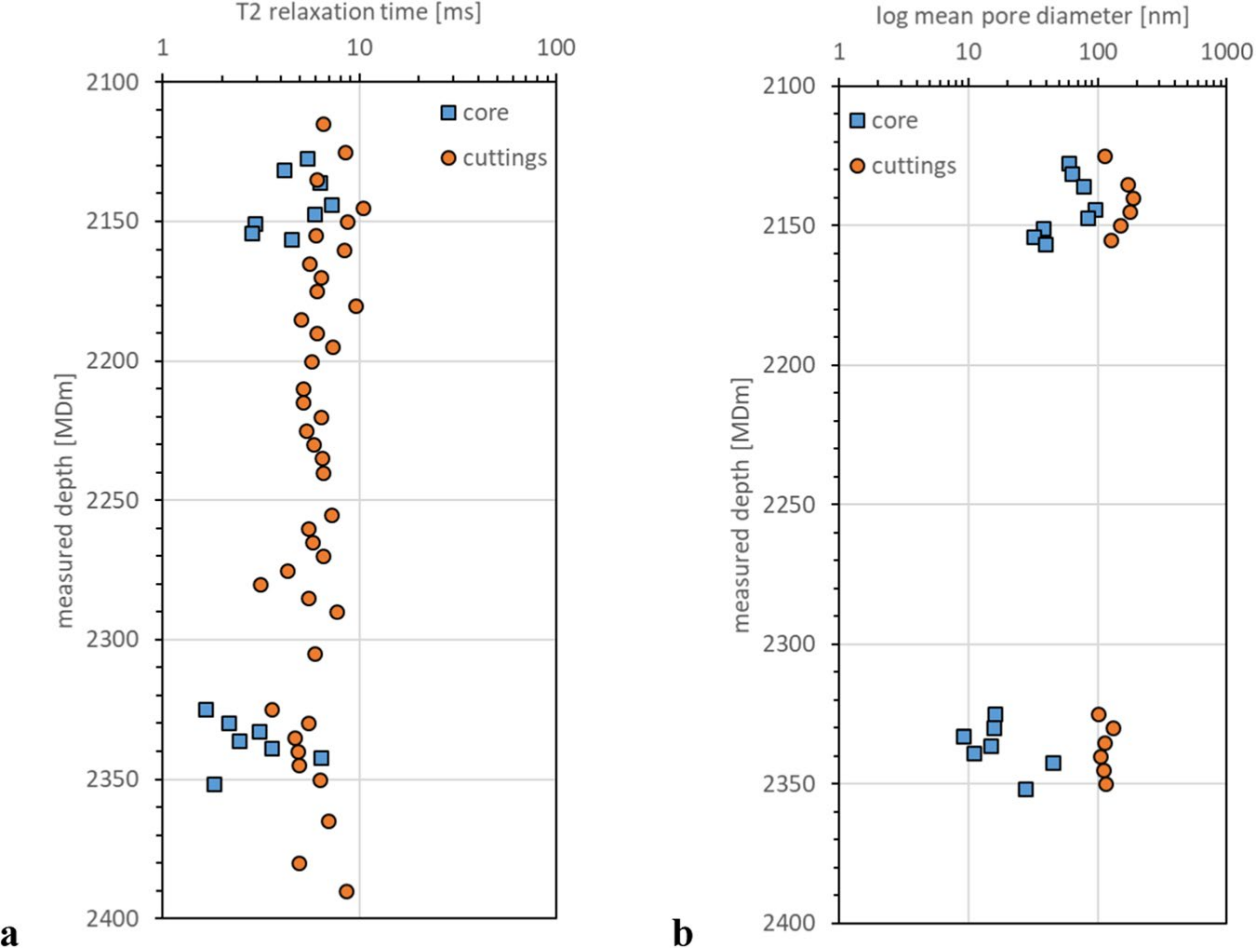
Depth distribution of pore throat size and NMR relaxation time. (**a**) Comparison of T2 relaxation time obtained with NMR measurements on solvent-cleaned cuttings (SCcutt) and solvent-cleaned core (SCcore) samples as a function of measured depth. (**b**) Comparison of logarithmic mean pore throat diameters obtained with MICP measurements on SCcutt and SCcore samples as a function of measured depth.

To evaluate the pore size of rock samples, which can be related to their permeability^11,12^, nuclear magnetic resonance (NMR) and mercury intrusion capillary pressure (MICP) measurements were performed. Figure 7a shows the comparison of T2 relaxation time obtained with NMR measurements on SCcutt and SCcore samples. Although the SCcutt samples show consistently, higher T2 relaxation time than that obtained with SCcore samples, both types of samples show similar trends. The Upper Montney Formation (2120 to 2160 m) has higher T2 relaxation time indicating larger pore diameters, whereas the Lower Montney Formation (2320 to 2360 m) has lower T2 relaxation time indicating smaller pore diameters.

Mean pore throat diameters obtained with MICP on the SCcutt and SCcore samples are shown in Fig. 7b. Similar to the NMR measurements, pore diameters obtained from MICP for the SCcutt samples show consistently larger pore diameters than those obtained from the SCcore samples. The overall trend, with larger pore diameters in the Upper Montney and smaller in the Lower Montney, remains consistent.

In both the NMR and MICP measurements, pore size evaluated with SCcutt samples indicated larger pore diameters than those obtained with ARcore samples. As discussed in the organic petrology section, the existence of drilling-induced micro-fractures in the cuttings likely results in overestimation of pore diameter.

## Conclusions

This study presents a comparative geochemical, petrographic, and petrophysical analysis of core samples and cuttings from the Montney tight gas siltstone reservoir. The results of bulk geochemistry show that, on average, the quantity of kerogen/solid bitumen measured in the as–received cuttings (ARcutt) samples using the extended slow heating (ESH) pyrolysis produce comparable results to that of the as-received core (ARcore) samples. The total organic carbon (TOC) measurements using the standard Rock-Eval analysis are influenced by OBM contamination. Solvent cleaning of cuttings can effectively remove OBM contamination in light, medium, and heavy range hydrocarbons and produce similar TOC quantification to that of core samples. However, the ESH method provides a faster and more economical alternative to the solvent cleaning of ARcutt samples.

Organic petrology further substantiates the presence of contamination by oil-based drilling mud as well as mud flocculates and caved fragments from shallower depths. These contaminations potentially influence the bulk geochemistry of ARcutt samples. Furthermore, silt grains in the cuttings appear to display widespread drilling-induced micro-fractures. Although the relative trend of pore throat size was captured with the measurements from cuttings samples, they resulted in overestimation of pore throat size compared with those from core samples. The observed overestimations in average pore size and pore throat diameter measured by NMR and MICP, respectively.

## Methods

Approximately 50 mg of dried and ground samples were used for pyrolysis analysis performed on a Rock-Eval 6 analyzer (Vinci Technologies) at the Geological Survey of Canada (GSC)^1,4,6,7,13–15^. Accuracy and precision of Rock-Eval pyrolysis parameters were determined using the GSC-9107 internal reference standard. This standard was analyzed prior to each new batch of samples and at least once every five samples to ensure consistency of measurements. Measured parameters have an accuracy and precision which deviated by less than 3% from the reference standard, with the exception of S3, which deviated by 8% from the standard value due to the high carbonate content of the standard.

Approximately 60 mg of powdered rock sample was subjected to online thermal desorption/pyrolysis-gas chromatography-mass spectrometry/flame ionization detection (TD/Py-GC-MS/FID) analysis using a Frontier EGA/PY-3030D pyrolyzer interfaced to an Agilent GC-MSD/FID dual detection system^1,6^. In both thermal desorption and pyrolysis modes, the volatile and semi-volatile hydrocarbons are thermally released into the pyrolyzer furnace and immediately carried away by a flow of helium carrier gas. The aforementioned hydrocarbons are then cryo-trapped at −186 °C at the head of the capillary column of the GC-MSD/FID system. Upon completion of thermal desorption or pyrolysis, the GC-MSD/FID analysis was initiated. The cryo-trapped hydrocarbons previously released into the GC column are molecularly separated based on their boiling points. The capillary column used for GC analysis was a PONA 50 m × 0.20 mm × 0.5 um, and helium was used as the carrier gas at a constant flow rate of 1 mL/min. GC oven temperature was initially held at 33 °C for 10 minutes, and subsequently raised to 63 °C at a rate of 3 °C/min, then at 6 °C/min to the final temperature of 325 °C where it was held for 35 minutes. At the end of the GC column, the eluent was split and fed into both MSD and FID detectors for dual detection. The MSD detector was used for compound identification while the FID detector was used for quantification. Compound identification was based on comparison of GC retention time and mass spectra with those identified in the literature.

The microscopic organic petrology study was carried out on selected ARcutt and ARcore samples using polished blocks made with a cold-setting epoxy-resin mixture. The resulting sample pellets were ground and polished in final preparation for microscopy using an incident light Zeiss Axioimager II microscope system equipped with both white and fluorescent light sources and the Diskus-Fossil system for image acquisition^1,4,6,7,14,15^.

Petrophysical analysis of samples was conducted using a nuclear magnetic resonance (NMR) instrument at the Japan Oil, Gas and Metals National Corporation (JOGMEC) Technology and Research Center. The NMR-T2 relaxation time was measured at 0.15 ms of the echo spacing for both drill cuttings and core samples. An ARcore plug with 1-inch diameter and 2-inch length extracted from a full diameter core was used. Solvent-cleaned cuttings (SCcutt) were used for NMR measurements on cuttings samples. Before NMR measurements, both sample sets were cleaned with toluene and methanol (24 hours for each fluid) and oven-dried at 100 °C for 24 hours. Then the samples were saturated with saline water (10,000 ppm) by applying 13,790 kPa (2000 psi) of forced injection after vacuuming. This procedure has been confirmed to sufficiently resolve the small pores (10 to 100 nm pore throat diameter) generally observed in previously studied Montney samples^16^.

Mercury injection capillary pressure (MICP) measurements were conducted using an AutoPore IV 9500 instrument provided by Micrometrics, which applies 413,700 kPa (60 000 psi) of maximum injection pressure corresponding to about 3 nm in pore throat diameter. The measurements on core samples were performed on crushed rock samples prepared from the end trims of the core plug samples used for the NMR measurements, while solvent-cleaned cuttings (SCcutt) were used for the measurements on cuttings. The same cleaning and drying procedure described above for NMR sample preparation were applied to both cuttings and core samples.

## Data Availability

The data that support the findings of this study are available from the corresponding author upon reasonable request.

## References

[1] Sanei H (2014). Petrographic and geochemical composition of kerogen in the Furongian (U. Cambrian) Alum Shale, central Sweden: Reflections on the petroleum generation potential. International Journal of Coal Geology.

[2] Ghanizadeh, A. *et al*. Petrophysical and geomechanical characteristics of Canadian tight oil and liquid-rich gas reservoirs: I. Pore network and permeability characterization. *Fuel* **153**, 664–681(2015a).

[3] Ghanizadeh, A. *et al*. Petrophysical and geomechanical characteristics of Canadian tight oil and liquid-rich gas reservoirs: II. Geomechanical property estimation. *Fuel* **153**, 682–691 (2015b).

[4] Aviles MA (2019). Organic petrography and geochemical characterization of the Upper Cretaceous Second White Specks and Upper Belle Fourche alloformations, west-central Alberta: Analysis of local maturity anomalies. International Journal of Coal Geology.

[5] Dewing K, Sanei H (2009). Analysis of large thermal maturity datasets: Examples from the Canadian Arctic Islands. International Journal of Coal Geology.

[6] Sanei H (2015). Characterization of organic matter fractions in an unconventional tight gas siltstone reservoir. International Journal of Coal Geology.

[7] Wood JM (2015). Solid bitumen as a determinant of reservoir quality in an unconventional tight gas siltstone play. International Journal of Coal Geology.

[8] Bustin, R. M. *et al*. Quantification of the gas- and liquid-in-place and flow characteristics of shale and other fine-grained facies in northeastern British Columbia; in Geoscience BC Summary of Activities 2014, Geoscience BC, Report 2015-1, p. 89–94 (2015).

[9] Wood, J. M. *et al*. Solid bitumen in the Montney Formation: Diagnostic petrographic characteristics and significance for hydrocarbon migration. *International Journal of Coal Geology* **198**, 48–62 (2018a).

[10] Wood, J. M. *et al*. Organic petrography and scanning electron microscopy imaging of a thermal maturity series from the Montney tight-gas and hydrocarbon liquids fairway. *Bulletin of Canadian Petroleum Geology* **66**, 499–515 (2018b).

[11] Sanei H (2015). Effects of nanoporosity and surface imperfections on solid bitumen reflectance (BRo) measurements in unconventional reservoirs. International Journal of Coal Geology.

[12] Akihisa K (2018). Integrating mud gas and cuttings analyses to understand local CGR variation in the Montney tight gas reservoir. International Journal of Coal Geology.

[13] Ardakani OH (2018). Do all fractions of organic matter contribute equally in shale porosity? A case study from Upper Ordovician Utica Shale, southern Quebec, Canada. Marine and Petroleum Geology.

[14] Kondla D (2015). Depositional environment and hydrocarbon potential of the Middle Triassic strata of the Sverdrup Basin, Canada. International Journal of Coal Geology.

[15] Van de Wetering N (2016). Organic matter characterization in mixed hydrocarbon producing areas within the Duvernay Formation, Western Canada Sedimentary Basin, Alberta. International Journal of Coal Geology.

[16] Akai T, Wood JM (2018). Application of pore throat size distribution data to petrophysical characterization of Montney tight-gas siltstones. Bulletin of Canadian Petroleum Geology.

